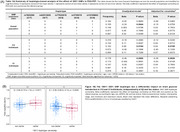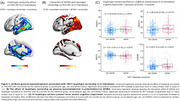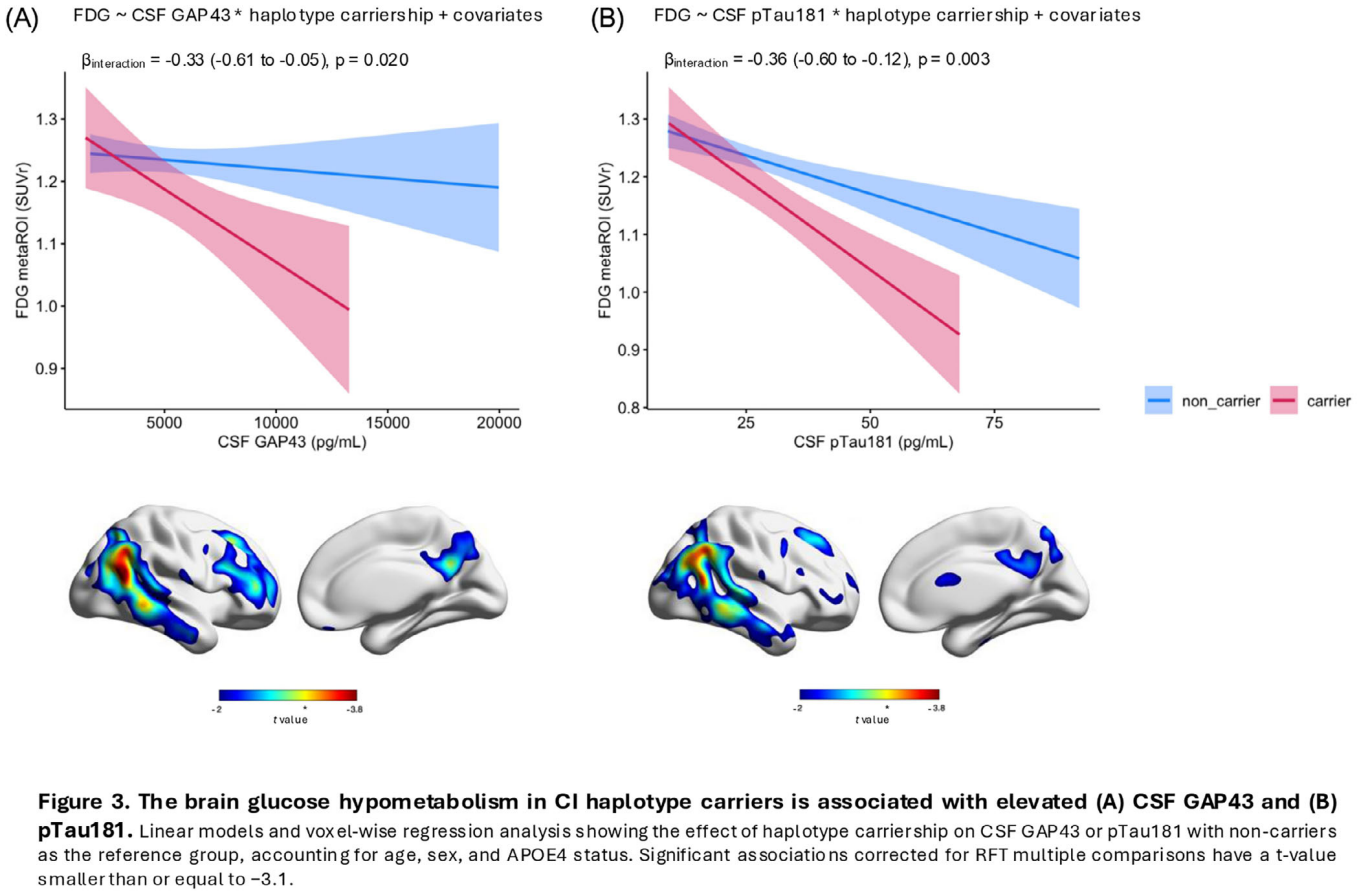# A common genetic variant of IDO1 exacerbates brain hypometabolism and cognitive decline

**DOI:** 10.1002/alz70856_100479

**Published:** 2025-12-25

**Authors:** Christian Limberger, João Pedro Ferrari‐Souza, Marco Antônio De Bastiani, Pedro Rosa‐Neto, Eduardo R. Zimmer

**Affiliations:** ^1^ Universidade Federal do Rio Grande do Sul, Porto Alegre, Rio Grande do Sul, Brazil; ^2^ McGill Centre for Studies in Aging, Montreal, QC, Canada; ^3^ Brain Institute of Rio Grande do Sul (InsCer), PUCRS, Porto Alegre, Rio Grande do Sul, Brazil

## Abstract

**Background:**

The enzyme indoleamine‐2,3‐dioxygenase (*IDO1*) is highly expressed in astrocytes in response to inflammatory stimuli associated with Alzheimer's disease (AD). *IDO1* plays a critical role in the conversion of tryptophan to kynurenine, a process that facilitates immune suppression under inflammatory conditions. Recent studies have revealed a novel role for *IDO1* in brain metabolism, showing that its activity impairs astrocytic glycolysis and reduces metabolic support to neurons in preclinical models of AD, thereby exacerbating amyloid and tau pathologies. However, the functional implications of gain‐ or loss‐of‐function single nucleotide polymorphisms (SNPs) in the *IDO1* gene among individuals with AD remain poorly understood. This study investigates whether *IDO1* SNP carriership contributes to metabolic disruptions across the AD continuum.

**Methods:**

We assessed 619 cognitively unimpaired (CU) and impaired (CI) individuals from ADNI with available baseline FDG‐PET, *IDO1* genotyping, CSF biomarkers, and cognitive assessments. SNP haplotype assignment was conducted for the combination of five identified *IDO1* SNPs (rs7820268, rs62512635, rs77622610, rs3739319, rs10108662). Generalized linear mixed‐models were performed to assess the effect of haplotype carriership on FDG‐PET for the five most frequent haplotypes. The haplotype with a significant impact on brain metabolism was subsequently tested for its interaction with APOE4 and association with cognitive decline or CSF biomarkers using linear models and voxel‐wise regression analysis (*p* <0.05).

**Results:**

The most frequent *IDO1* SNP haplotype (15.5%) corresponds to carriers of one minor allele for rs7820268, rs3739319, and rs10108662, referred to here as the 10011 haplotype (Table 1A). Carriership of this haplotype was associated with distinct alterations in brain glucose metabolism, characterized by hypermetabolism in CU and hypometabolism in CI individuals, independent of Aβ and tau status (Figure 1B). CI carriers of the 10011 haplotype exhibited prominent hypometabolism in the temporal region (Figure 2A), an effect potentialized by APOE4 (Figure 2B). In addition, CI carriers presented severe cognitive impairment, as confirmed by four different assessments (Figure 2C). Finally, glucose hypometabolism was associated with higher levels of CSF pTau181 and GAP43 (Figure 3A‐B).

**Conclusion:**

To the best of our knowledge, this is the first in vivo clinical evidence demonstrating that an *IDO1* SNP exacerbates brain hypometabolism and worsens cognitive decline in AD.